# Cleavage of Fibulin-2 by the aggrecanases ADAMTS-4 and ADAMTS-5 contributes to the tumorigenic potential of breast cancer cells

**DOI:** 10.18632/oncotarget.14627

**Published:** 2017-01-11

**Authors:** Tania Fontanil, Saúl Álvarez-Teijeiro, M. Ángeles Villaronga, Yamina Mohamedi, Laura Solares, Angela Moncada-Pazos, José A. Vega, Olivia García-Suárez, Marcos Pérez-Basterrechea, Juana M. García-Pedrero, Alvaro J Obaya, Santiago Cal

**Affiliations:** ^1^ Departamento de Bioquímica y Biología Molecular, Universidad de Oviedo, Asturias Spain; ^2^ Instituto Universitario de Oncología, IUOPA, Universidad de Oviedo, Asturias, Spain; ^3^ Department of Morphology and Cellular Biology, Faculty of Medicine, University of Oviedo, Oviedo, Spain; ^4^ Hospital Universitario Central de Asturias, Universidad de Oviedo, Asturias, and CIBERONC, Madrid, Spain; ^5^ Facultad de Ciencias de la Salud, Universidad Autónoma de Chile, Chile; ^6^ Unidad de Trasplantes, Terapia Celular y Medicina Regenerativa, Hospital Universitario Central de Asturias, Oviedo, Spain; ^7^ Departamento de Biología Funcional, Area de Fisiología, Universidad de Oviedo, Asturias, Spain; ^8^ Sir William Dunn School of Pathology, University of Oxford, Oxford OX1 3RE, United Kingdom

**Keywords:** extracellular matrix, aggrecanases, ADAMTS, metalloproteases, fibulin

## Abstract

Fibulin-2 participates in the assembly of extracellular matrix components through interactions with multiple ligands and promotes contacts between cells and their surrounding environment. Consequently, identification of processes that could lead to an altered Fibulin-2 could have a major impact not only in the maintenance of tissue architecture and morphogenesis but also in pathological situations including cancer. Herein, we have investigated the ability of the secreted metalloproteases ADAMTS-4 and ADAMTS-5 to digest Fibulin-2. Using *in vitro* approaches and cultured breast cancer cell lines we demonstrate that Fibulin-2 is a better substrate for ADAMTS-5 than it is for ADAMTS-4. Moreover, Fibulin-2 degradation is associated to an enhancement of the invasive potential of T47D, MCF-7 and SK-BR-3 cells. We have also found that conditioned medium from MCF-7 cells that simultaneously overexpress Fibulin-2 and ADAMTS-5 significantly induced the migratory and invasive ability of normal breast fibroblasts using 3D collagen matrices. Immunohistochemical analysis highlights the close proximity or partial overlap of both Fibulin-2 and ADAMTS-5 in breast tumor samples. Additionally, proteolytic products derived from a potential degradation of Fibulin-2 by ADAMTS-5 were also identified in these samples. Finally, we also show that the cleavage of Fibulin-2 by ADAMTS-5 is counteracted by ADAMTS-12, a metalloprotease that interacts with Fibulin-2. Overall, our results provide direct evidence indicating that Fibulin-2 is a novel substrate of ADAMTS-5 and that this proteolysis could alter the cellular microenvironment affecting the balance between protumor and antitumor effects associated to both Fibulin-2 and the ADAMTSs metalloproteases.

## INTRODUCTION

Fibulins are a family of seven secreted glycoproteins characterized by a complex molecular architecture [1, 2]. All members of this family share a variable number of EGF-like domains at their central portion and a fibulin-type module at the C-terminal region of the protein. Domains at the N-terminal region are the most variable among all members and, in the cases of Fibulin-1 and Fibulin-2, three anaphylatoxin domains can be identified. Additionally, Fibulin-2 possesses two distinctive domains called Cys-rich and Cys-free segments within its N-terminal region. This broad variety of domains serves to establish interactions with multiple ECM and cell surface components. For instance, Fibulin-2 can interact with aggrecan [3], nidogen [4], fibronectin [5], and perlecan [6] among others, as well as cellular integrins [7], thus contributing to the maintenance of extracellular structures such as basement membranes and elastic fibers [1]. However, Fibulin-2 has also been involved in functions other than its structural role at the ECM. Thus, Fibulin-2 is rapidly upregulated during skin wound healing suggesting its participation in cell mobility and tissue repair processes [8]. In addition, a growing number of studies highlights the involvement of Fibulin-2 in tumorigenesis. In this regard, Fibulin-2 can display both tumor-promoting [9, 10] and tumor-protective [11, 12] properties in different types of neoplasia. Mechanisms underlying these opposing effects are complex and not fully understood [13, 14].

Functional relevance of Fibulin-2 makes it paramount to identify and understand those situations in which the function of this secreted glycoprotein is altered. In this respect, previous works have revealed that Fibulin-2 can be proteolytically processed by MMPs and serine proteases involved in tissue remodeling processes [15], whereas its closest partner Fibulin-1 is more resistant to this proteolysis. Cleavage of Fibulin-2 can occur not only during normal metabolism but also associated to pathological processes. As an example, it is known that Fibulin-2 is highly expressed in blood vessels and protects from vascular injuries, so it is possible that this glycoprotein could be degraded by proteases in vascular lesions [15, 16]. It has been also found that Fibulin-2 is degraded in photodamaged skin, which is attributed to an increase in the proteolytic activity [17]. Fibulin-2 degradation has also been associated to tumorigenesis in osteosarcoma [18]. In fact, a considerable amount of Fibulin-2 proteolytic products are detected in osteosarcoma cell lines in comparison with primary osteoblasts. Gelatinases MMP-2 and MMP-9 are responsible for Fibulin-2 degradation in these tumor cells. Furthermore, susceptibility of Fibulin-2 to the degradation mediated by MMP-2 has been confirmed through *in vitro* biochemical approaches [19]. By contrast, Fibulin-1 is also highly resistant to the proteolysis mediated by this gelatinase [18].

ADAMTS-4 and ADAMTS-5 are members of the ADAMTS family of secreted metalloproteases [20]. Both enzymes are also known as aggrecanases owing their ability to cleave cartilage aggrecan [21]. Additionally, aggrecanases can also degrade ECM components other than aggrecan, such as brevican [22], biglycan [23], versican [24], a_2_-macroglobulin [25] or matrilin-2 [26]. This broad spectrum of substrates highlights the importance of ADAMTS-4 and ADAMTS-5 in key physiological processes [27, 28], as well as in pathological disorders including osteoarthritic diseases [29] and cancer [20, 30]. In this work we show that the aggrecanases, mainly ADAMTS-5, can cleave Fibulin-2 both *in vitro* and in cultured breast cancer cell lines. Moreover, Fibulin-2 digestion increases the tumorigenic potential of the poorly invasive T47D and MCF-7 cell lines. We have also investigated the localization of both Fibulin-2 and ADAMTS-5 in breast cancer samples as well as the effect that the conditioned medium of breast cancer cells that exogenously express Fibulin-2 alone or in combination with ADAMTS-5 produces on normal mammary fibroblasts. In addition, we have also found that Fibulin-2 degradation by ADAMTS-5 can be blocked by ADAMTS-12, another member of the ADAMTS family of metalloproteases that interacts with and enhances the antitumor effects mediated by Fibulin-2 [31]. Our data strongly suggest that the cleavage by aggrecanases, but especially by ADAMTS-5, could influence the balance between pro- and anti-tumor effects elicited by Fibulin-2.

## RESULTS

### Proteolytic digestion of Fibulin-2 by aggrecanases

**Figure 1:**
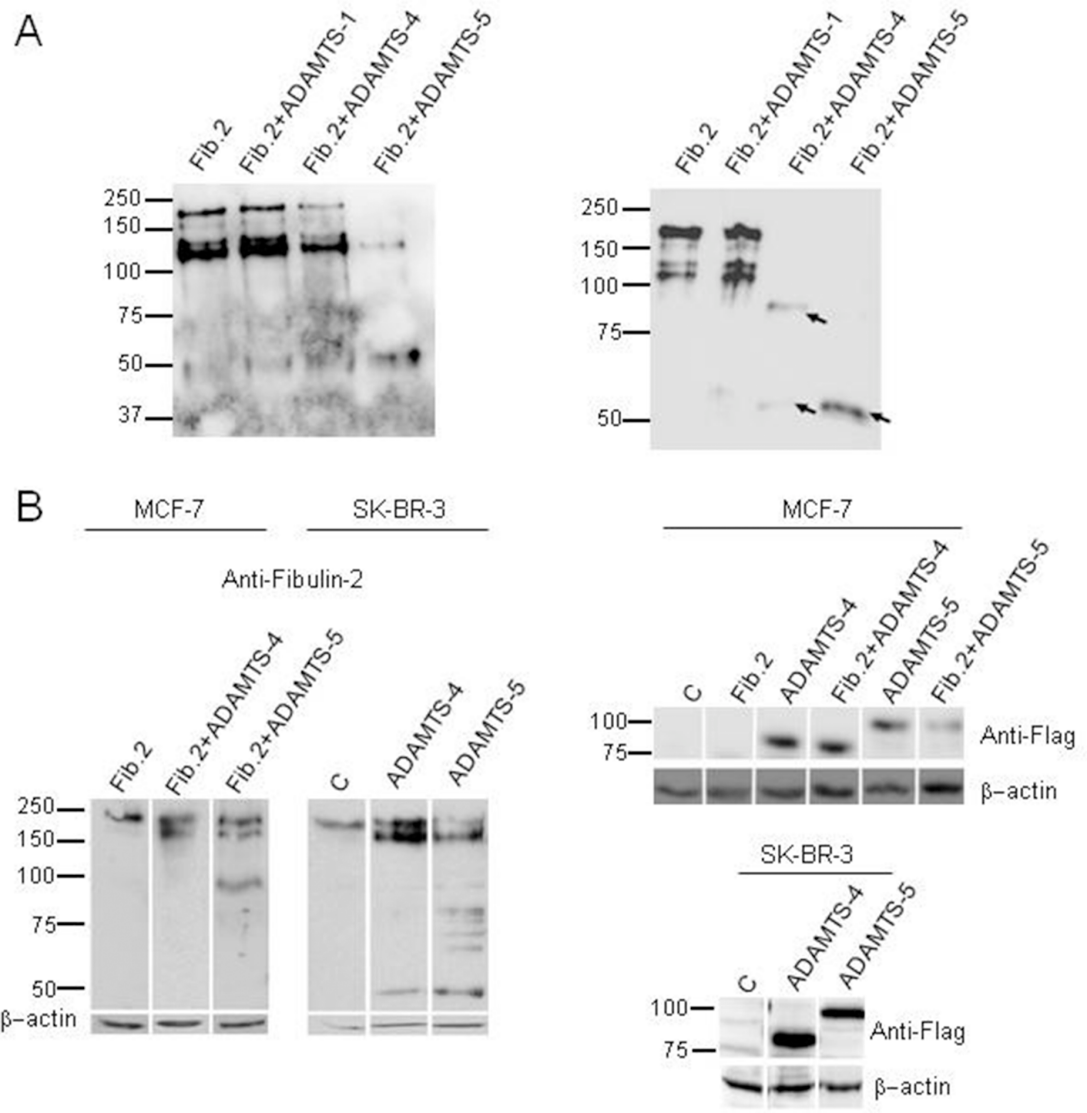
Fibulin-2 cleavage by ADAMTS-4 and ADAMTS-5 **(A)** Left, in vitro digestion of Fibulin-2 by aggrecanases. Fibulin-2 was incubated with ADAMTS-1 (Fib.2 + ADAMTS-1), ADAMTS-4 (Fib.2 + ADAMTS4) or ADAMTS-5 (Fib.2 + ADAMTS-5) for 16 h. Fib.2 indicates Fibulin-2 incubated alone (without metalloproteases, control). Right, an independent experiment was perfomed to evaluate final degradation products. To this end, Fibulin-2 was incubated for 24 h with ADAMTS-1 (Fib.2 + ADAMTS-1), ADAMTS-4 (Fib.2 + ADAMTS-4) or ADAMTS-5 (Fib.2 + ADAMTS-5). Main proteolytic products were detected with an anti-Fibulin-2 antibody and are indicated with arrows. Molecular weight markers are indicated on the left. **(B)** Left, MCF-7 cells were transfected with a plasmid containing the full-length cDNA for Fibulin-2 (Fib.2) or co-transfected with this plasmid and plasmids containing the full-length cDNA for ADAMTS-4 (Fib.2 + ADAMTS-4), or ADAMTS-5 (Fib.2 + ADAMTS-5). SK-BR-3 produces fibulin-2 endogenously and they were transfected with an empty vector (C, Control), or with vector containing the full-length cDNAs for ADAMTS-4 or ADAMTS-5. Right, expression of exogenous ADAMTS-4 and ADAMTS-5 was analyzed by using an anti-FLAG antibody (bottom panels). Intervening irrelevant lanes are not shown. Molecular weight markers are indicated on the left and β-actins were used as loading control.

As part of our work aimed at identifying new interactions between ADAMTS metalloproteases and ECM components, we found that Fibulin-2 is a novel substrate for the ADAMTS-4 and ADAMTS-5 metalloproteases. As can be seen in Figure 1A, incubation of Fibulin-2 with these proteolytic enzymes resulted in its degradation. This analysis also suggested that ADAMTS-5 could be more effective than ADAMTS-4 at cleaving Fibulin-2 attending to the reduced intensity of the major immunoreactive band that corresponds to the entire Fibulin-2 and to the accumulation of a main proteolytic product of about 50 kDa using same enzyme concentrations and incubation times. In this assay, we also wanted to evaluate whether ADAMTS-1 was also able to degrade Fibulin-2 taking into account that ADAMTS-1, ADAMTS-4 and ADAMTS-5 share the ability to digest not only aggrecan but also other hyalectans such as versican or neurocan [32]. However, Fibulin-2 was not cleaved by ADAMTS-1 under the same experimental conditions (Figure 1A). We also examined whether the 50 kDa band could be the final degradation product. Following 24 h incubation with either ADAMTS-4 or ADAMTS-5 entire Fibulin-2 was not detected and the 50 kDa band was the only detectable band by ADAMTS-5 proteolysis and one of the main proteolytic products by ADAMTS-4 proteolytic activity (Figure 1A). These data would indicate that the 50 kDa fragment is the consequence of a specific proteolysis rather than the result of a non-specific degradation by the aggrecanases.

Next, we examined the possibility that the cleavage of Fibulin-2 mediated by aggrecanases could occur in cultured cells. To this end, we used MCF-7 cells, a breast cancer cell line that does not express Fibulin-2 [12]. Following co-transfection with a vector containing the full-length cDNA for Fibulin-2 together with a vector containing the full-length cDNA for either ADAMTS-4 or ADAMTS-5, cell extracts were analyzed by western blot, and we found that Fibulin-2 resulted proteolytically cleaved in the presence of the aggrecanases but especially by ADAMTS-5 (Figure 1B).

To elucidate whether endogenous Fibulin-2 could also be degraded by these proteases, we carried out transfections only with plasmids containing the full-length cDNA for ADAMTS-4 or ADAMTS-5 into SK-BR-3 breast cancer cells, which endogenously express Fibulin-2 [12]. Results again highlighted the ability of both aggrecanases to digest Fibulin-2, particularly ADAMTS-5 (Figure 1B). These data suggest that the proteolysis of Fibulin-2 by these metalloproteases may also occur *in vivo*.

MCF-7 cells were also employed to ascertain that ADAMTS-1 does not digest Fibulin-2 in culture cells. Following co-transfection with the corresponding cDNAs our result revealed that Fibulin-2 remains mostly intact as assessed by western blot (Supplementary Figure 1A). In addition, we employed HEK293 cells attending to their high efficiency of transformation to examine whether Fibulin-2 degradation products can be detected as soluble fragments in cell conditioned medium. Western blot analysis pointed out the presence of immunoreactive bands in conditioned medium from HEK293 cells co-transfected with cDNAs for Fibulin-2 and ADAMTS-5, which were absent in the medium from the same cell line transfected with only cDNA for Fibulin-2 (Supplementary Figure 1B).

### Proteolysis of Fibulin-2 by aggrecanases increases the invasive phenotype of MCF-7 and T47D breast cancer cell lines

**Figure 2:**
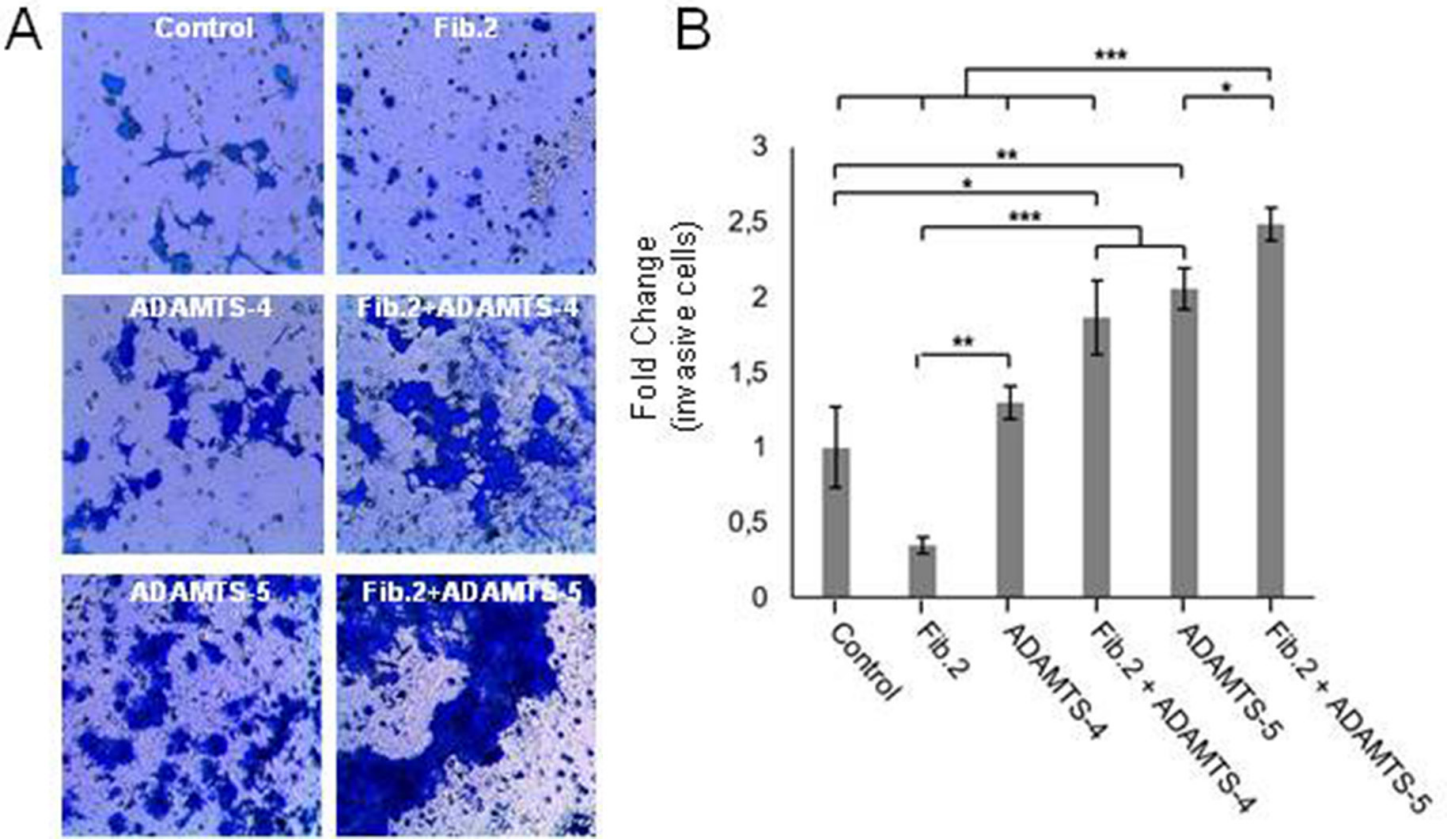
Invasion capacity of MCF-7 is increased by the simultaneous presence of Fibulin-2 and ADAMTS-5 **(A)** Representative pictures showing invasive MCF-7 cells using Matrigel-coated invasion chambers. Control, MCF-7 cells transfected with an empty vector. **(B)** Quantification of fold-change in invasion capacity of MCF-7 cells as compared with control cells (relative values) under the different conditions assayed.

To examine whether the degradation of Fibulin-2 could alter the invasive potential of MCF-7 cells, we performed cell invasion assays using Matrigel-coated invasion chambers with MCF-7 cells expressing Fibulin-2 alone (MCF-7 Fib.2) or in combination with either ADAMTS-4 (Fib.2+ADAMTS-4) or ADAMTS-5 (Fib.2+ADAMTS-5). MCF-7 cells that express ADAMTS-4 alone or ADAMTS-5 alone were employed for comparative purposes, and MCF-7 transfected with an empty vector were employed as control (Figure 2A and 2B). Results revealed that MCF-7 Fib.2+ADAMTS-5 cells showed the highest capacity to invade (2.5-fold increase as compared with control cells), followed by MCF-7 ADAMTS-5 (2.0-fold), MCF-7 Fib.2 + ADAMTS-4 (1.9-fold) and MCF-7 ADAMTS-4 (1.3-fold). By contrast, presence of Fibulin-2 alone considerably reduces the ability as of MCF-7 cells (MCF-7 Fib.2 cells) to invade through the Matrigel-coated invasion chambers. Similar results were obtained using T47D cells, other breast cancer cell line that does not express Fibulin-2 [12]. Following transfection with the corresponding full-length cDNAs to express the indicated recombinant proteins (Supplementary Figure 2A), we performed invasion assays to determine that T47D Fib.2+ADAMTS-5 cells showed the highest increase (3.4-fold), followed by T47D Fib.2+ADAMTS-4 (2.3-fold), T47D ADAMTS-5 (2.0-fold) and T47D ADAMTS-4 (1.9-fold) cells as compared with T47D control cells. Presence of Fibulin-2 alone impeded T47D cells to invade as T47D Fib.2 cells were barely detected in the lower surface of the invasion chamber (Supplementary Figure 2B). It was also noticeable the morphological change undergone by T47D cells expressing Fibulin-2 with ADAMTS-4 or ADAMTS-5. In fact, T47D is a cell line that exhibits an epithelial-like morphology [33, 34]. However simultaneous presence of Fibulin-2 and an aggrecanase, mainly ADAMTS-5, induced morphological changes in T47D cells from cobblestone-like to spindle-like shape (Supplementary Figure 2C). This effect has been associated to an epithelial-mesenchymal transition process in T47D cells [34]. Taking together, these results illustrate that Fibulin-2 can influence breast cancer cell behavior depending on the presence of aggrecanases.

### Effect of tumor cell-conditioned media on mammary fibroblast spheroids

**Figure 3:**
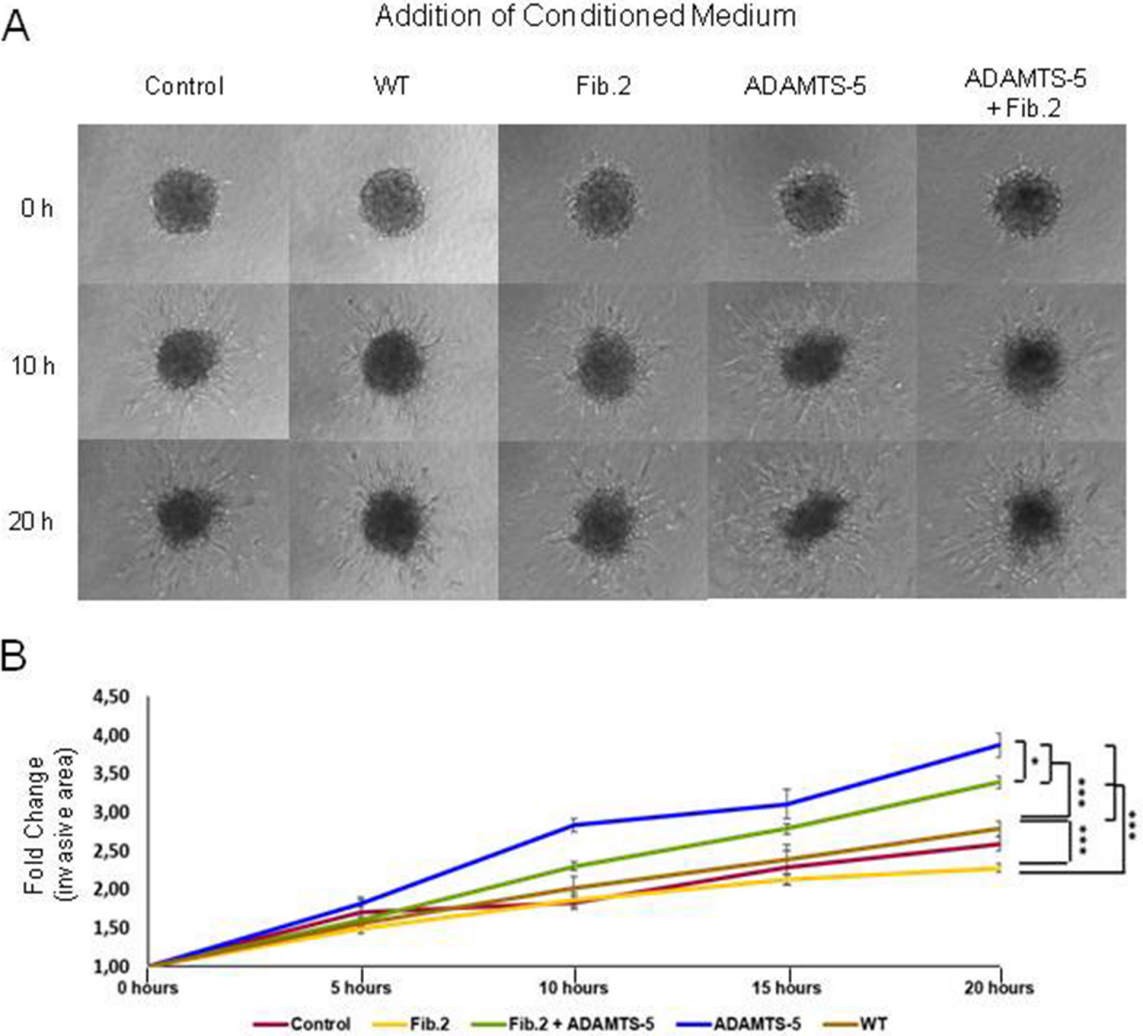
Effect of conditioned medium from MCF-7 cells on normal mammary fibroblasts spheroids in 3D collagen matrices **(A)** Representative images of fibroblast spheroids exposed to conditioned medium from MCF-7 cells transfected with an empty vector (WT) or cells overexpressing Fibulin-2 (Fib.2), ADAMTS-5 (ADAMTS-5) or both (Fib.2 + ADAMTS-5) at the indicated times. Medium without cultured cells was employed as control medium. **(B)** Measure of the branching (invasive area) by the fibroblasts spheroids as fold-changes increases at the indicated times.

Taking into account previous results showing that ADAMTS-5 induces more significant differences on breast cancer cells behavior than ADAMTS-4, we focused our further efforts on ADAMTS-5 through the examination of the effect of different conditioned media on normal mammary fibroblasts. We first verified that the mammary fibroblasts employed in this study produce Fibulin-2 (Supplementary Figure 3), as it has been previously demonstrated for human skin and embryonic fibroblasts [35]. Then different conditioned media from MCF-7 cells, which showed a low level of endogenous ADAMTS-5 expression among the three breast cancer cell lines employed in this work (Supplementary Figure 3), were added to normal mammary fibroblasts spheroids embedded in 3D collagen matrix (Figure 3A and 3B, and Supplementary Videos 1 to 5). Result revealed that the exposure of spheroids to conditioned medium from MCF-7 ADAMTS-5 cells induced an extensive branching (3.9-fold change at 20 hours) expanding into the surrounding matrix (invasive area). Fibroblasts spheroids exposed to conditioned medium from MCF-7 transfected with an empty vector or to medium alone (control condition) showed 2.8-fold and 2.6-fold increase in their invasive areas respectively; whereas conditioned medium from MCF-7 Fib.2 cells induced the lowest increase in the spheroids invasive area (2.2-fold) at the same incubation time. However, conditioned medium from MCF-7 cells that simultaneously expressed Fibulin-2 and ADAMTS-5 (MCF-7 Fib2+ADAMTS-5 cells) stimulated the increase of the invasive area 3,3-fold, value closer to that obtained for conditioned medium from MCF-7 ADAMTS-5 cells. Moreover, we found that this effect was accompanied by an increase in the α-SMA levels (Supplementary Figure 4), a hallmark of stromal fibroblasts isolated from breast cancer tissues [36]. These results suggest that the protective role of Fibulin-2 can be neutralized by its degradation by ADAMTS-5 affecting the migratory and invasive potential of the stromal fibroblasts.

### Detection of ADAMTS-5 and Fibulin-2 in breast cancer samples

**Figure 4:**
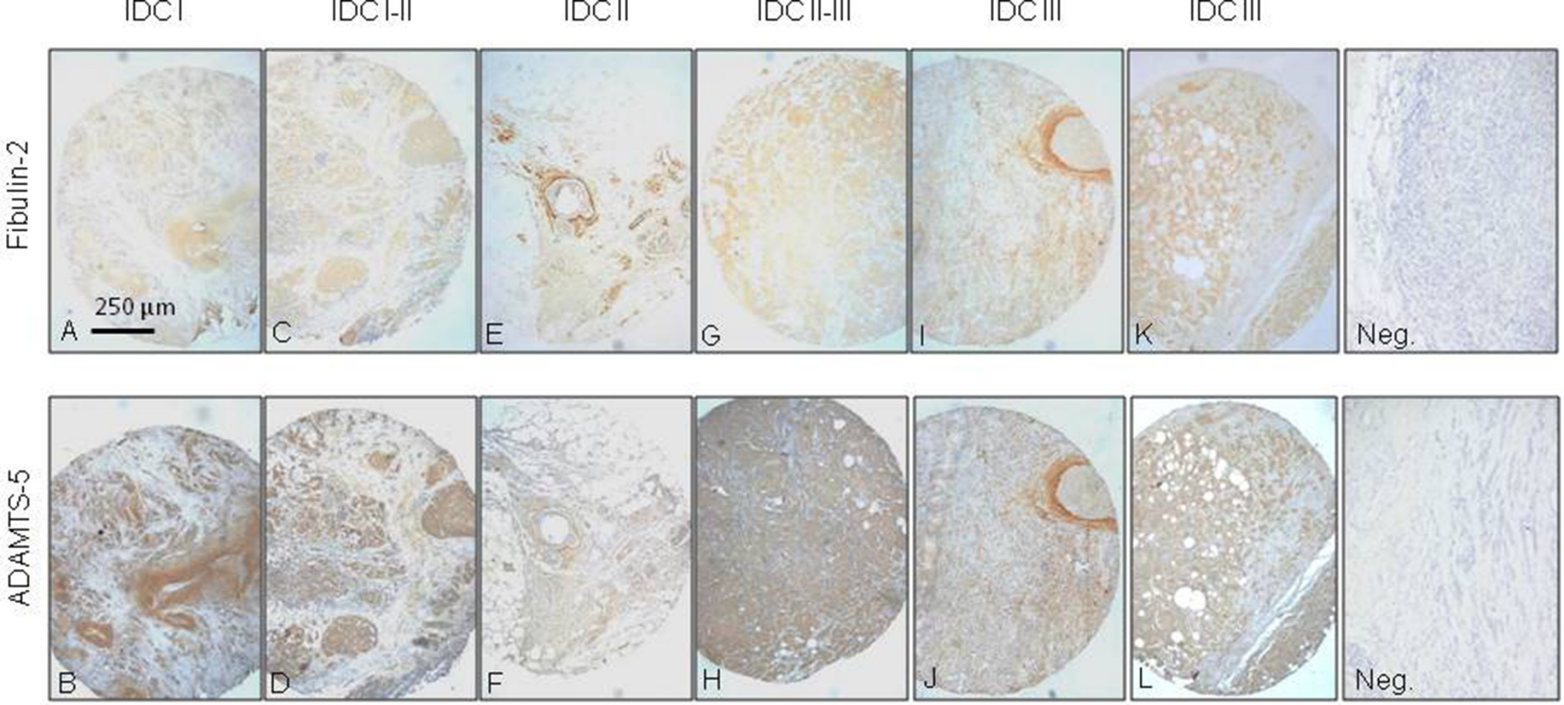
Immunohistochemical analysis of Fibulin-2 and ADAMTS-5 expression in breast cancer specimens Representative examples of invasive ductal breast carcinomas (IDC) at different stages (**A** and **B**, I; **C** and **D**, I–II; **E** and **F**, II; **G** and **H**, II–III; and **I**, **J**, **K** and **L**, III) showing staining for Fibulin-2 and ADAMTS-5. Negative controls (Neg.) are on the right.

We performed an immunohistochemical analysis of the expression of ADAMTS-5 and Fibulin-2 in a tissue array containing 48 invasive ductal carcinoma (IDC) tissue samples from different differentiation grades (14 samples IDC grade I, IDC-I; 6 grade I–II; 6 grade II; 4 grade II–III; and 18 grade III), as IDC is the most common type of breast cancer. Overall, our data indicate a close proximity between both proteins independently of the tumor stage using serial slides, but showing different intensities for each case. Some representative examples are indicated in Figure 4. For instance in a IDC-I sample, Fibulin-2 was faintly detected in large duct walls and periductal spaces (Figure 4A), whereas ADAMTS-5 immunostaining was also detected in large mammary ducts but showing a strong intensity (Figure 4B). In IDC I–II samples, Fibulin-2 was barely detected in tumor cell masses (Figure 4C) and ADAMTS-5 was present in masses of epithelial cells (Figure 4D). In IDC II and IDC II–III, Fibulin-2 was in the periductal and perivascular stroma (Figure 4E) or in some well delimited cells of the tumor cells masses (Figure 4G); whereas ADAMTS-5 showed a faint immunoreactivity paralleled to that of Fibulin-2 (Figure 4F) or diffusely presented in masses of epithelial cells (Figure 4H). In IDC III, a strong immunoreactivity for both Fibulin-2 (Figure 4I and 4K) and ADAMTS-5 (Figure 4J and 4L) was observed in the capsular stroma surrounding solid masses as well as diffusely distributed in the tissue stroma. Moreover, some tumor regions close to large blood vessels were encircled by ADAMTS-5 and Fibulin-2 positive cells.

**Figure 5:**
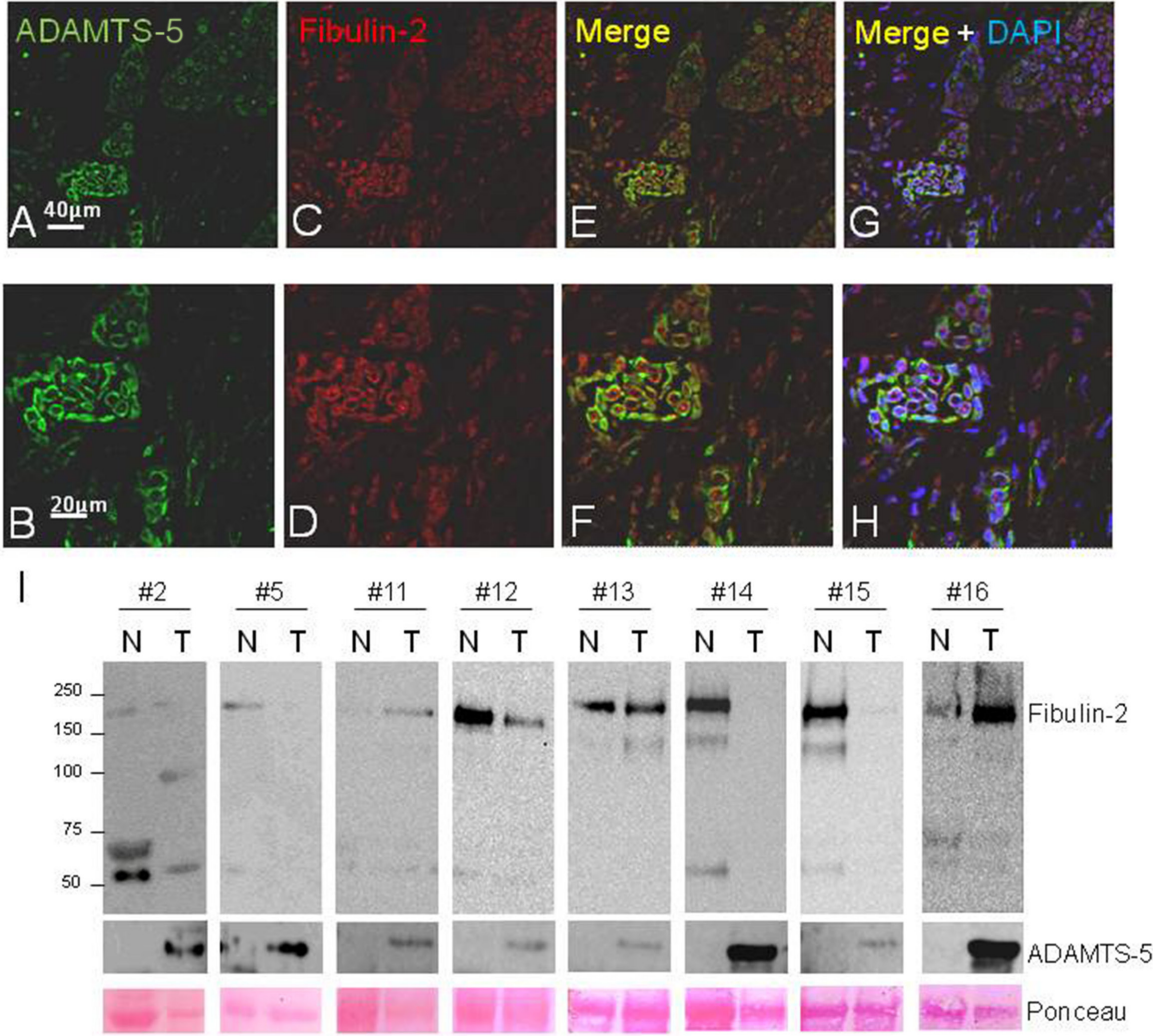
Fluorescent immunostaining of ADAMTS-5 and Fibulin-2 in invasive ductal breast carcinoma and western blot analysis of paired normal-tumor breast cancer tissue samples (**A** and **B**), detection of ADAMTS-5 (green); and (**C** and **D**), detection of Fibulin-2 (red). (**E** and **F**), merged images. (**G** and **H**), show merged images counterstained with DAPI to localize the nucleus. I, detection of Fibulin-2 and ADAMTS-5 by western blot in a set of eight matched normal (N) and tumor (T) breast cancer samples. (#2, grade I; #5 and #12, grade I–II; #11, #15 and #16, grade II; #13 and #14, grade III). Molecular weight markers are on the left and Ponceau S staining was employed to assess protein loading.

An immunofluorescence analysis to better examine the localization of both Fibulin-2 and ADAMTS-5 was also performed in IDC III (Figure 5). In this analysis ADAMTS-5 immunoreactivity was observed labeling the stroma (Figure 5A and 5B), whereas a faint but specific immunoreaction for Fibulin-2 was detected in the epithelial cell clusters (Figure 5C and 5D). When images where merged (Figure 5E to 5H) Fibulin-2 and ADAMTS-5 showed a close proximity or a partial but not a full co-localization.

Presence of Fibulin-2 and ADAMTS-5 was also examined by western blot in a panel of eight paired normal-tumor breast cancer samples with the finding that an immunoreactive band corresponding to the metalloprotease was detected in all tumor samples (Figure 5I). However, entire Fibulin-2 was mainly detected in normal tissues and an approximately 50 kDa immunoreactive band was also discernible in different tumor but also in normal samples. Six breast tumor samples corresponding to advanced stages of the disease were additionally analyzed and a weak signal corresponding to the entire Fibulin-2 was detected in three samples and ADAMTS-5 was identified in most of them (Supplementary Figure 5). These data would indicate that the cleavage of Fibulin-2 by ADAMTS-5 may also occur *in vivo*. However, other factors can influence the presence or function of both extracellular matrix proteins and it might be a proteolysis process also mediated by different proteases. As an illustrative approach we performed an *in vitro* digestion of Fibulin-2 with ADAMTS-5 or MMP-2 (Supplementary Figure 5). Following 4 or 10 hours of incubation, patterns of proteolytic digestion were analyzed by western blot with the finding that some of the immunoreactive bands could correspond to those identified in breast tumor samples according to their molecular weight. This result strongly suggests that Fibulin-2 cleavage in breast tumors can be mediated by different proteases.

### ADAMTS-12 hampers the degradation of Fibulin-2 by ADAMTS-5

**Figure 6:**
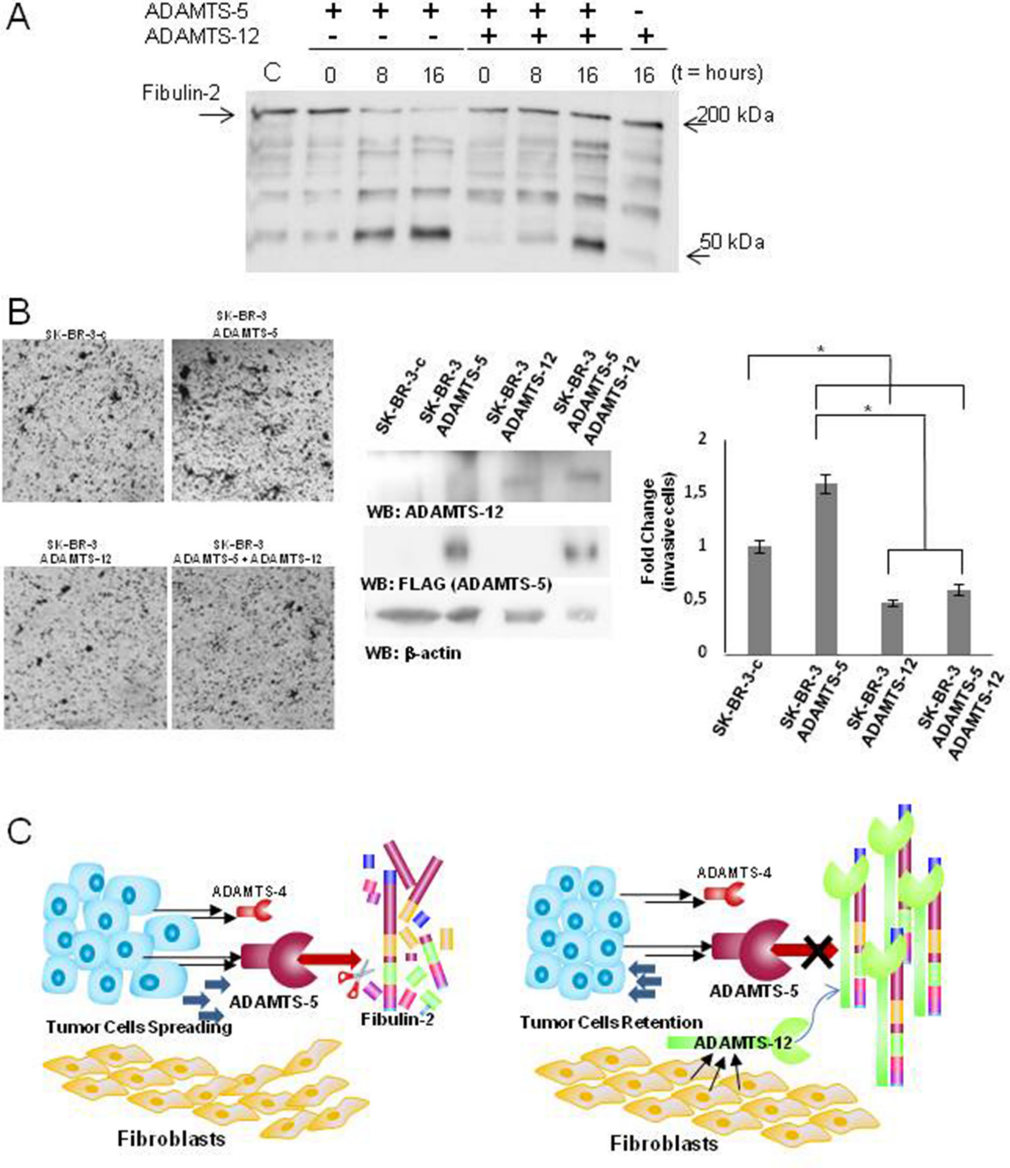
ADAMTS-12 blocks digestion of Fibulin-2 by ADAMTS-5 **(A)** Fibulin-2 was incubated with ADAMTS-5 in the presence (+) or absence (−) of ADAMTS-12 at 0, 8 and 16 h, and results were examined by western blot. **(B)** Presence of ADAMTS-12 decreases the ability of SK-BR-3 overproducing ADAMTS-5 to invade. Left, representative pictures showing the invasive SK-BR-3 cells using Matrigel-coated invasion chambers. SK-BR-3c indicates cells transfected with an empty vector; ADAMTS-5, SK-BR-3 cells expressing exogenous ADAMTS-5; ADAMTS-12 indicates cells expressing exogenous ADAMTS-12, and ADAMTS-5 + ADAMTS-12 indicates SK-BR-3 cells expressing both metalloproteases as is indicated in the center panel (β-actin was employed as loading control). Right, quantification of fold-change in invasion capacity of SK-BR-3 cells under the different conditions assayed. **(C)** Proposed models for the influence of the examined ADAMTS metalloproteases in the progression of breast cancer. Left, secretion of ADAMTS-4 and particularly of ADAMTS-5 by tumor cells can contribute to cleave Fibulin-2 thus leading to cancer cell spreading and an increase of the migratory potential of the fibroblasts. Right, ADAMTS-12 may act as a tumor-protective metalloprotease through the interaction with Fibulin-2. This interaction may impede digestion of Fibulin-2 by aggrecanases thus hindering progression of tumor cells.

We have previously reported that ADAMTS-12 is an interacting partner of Fibulin-2 and that this interaction promotes tumor-protective effects in breast cancer cells [31]. In the present work we wanted to analyze whether ADAMTS-12 could affect Fibulin-2 cleavage by ADAMTS-5. To this end we incubated Fibulin-2 with ADAMTS-5 in the presence or absence of ADAMTS-12. While the degradation of Fibulin-2 by ADAMTS-5 is clearly observed at 8 and 16 h of incubation in the absence of ADAMTS-12, its presence hinders Fibulin-2 proteolysis (Figure 6A). Also, accumulation of the proteolytic product around 50 kDa is already detected at 8 h of incubation in the absence of ADAMTS-12, but not in its presence. To investigate whether the presence of ADAMTS-12 could affect the behavior of breast cancer cells overexpressing ADAMTS-5, we carried out invasion assays using SK-BR-3 cells bearing in mind their endogenous expression of Fibulin-2. SK-BR-3 cells overexpressing ADAMTS-5 increased their ability to invade when compared with SK-BR-3 control cells (SK-BR-3c) (Figure 6B). However, exogenous expression of ADAMTS-12 did not only reduce the capacity of cells to invade but also counteracted the increase of the invasive capacity caused by ADAMTS-5. Taking into account these results, it could be hypothesized that ADAMTS-12 protects Fibulin-2 from the degradation mediated by ADAMTS-5, which could influence the equilibrium between tumor-promoting and tumor-preventing functions attributed to Fibulin-2 (Figure 6C).

## DISCUSSION

In this work we found that Fibulin-2 is a novel substrate of the aggrecanases, in particular of ADAMTS-5. This can be explained by the fact that ADAMTS-5 has a higher intrinsic catalytic ability than that of ADAMTS-4 [37]. In this sense, ADAMTS-5 shows much higher aggrecanolytic activity than ADAMTS-4 [38]. This observation could be of interest in relation to the tumor-protective and tumor-promoting effects associated to both Fibulin-2 [13, 14] and the ADAMTS metalloproteases [20, 30]. Thus, it can be hypothesized that cleavage of Fibulin-2 by ADAMTS-5 could form part of the strategies employed by tumor cells to degrade ECM components thereby facilitating tumor cell invasion. In this regard, degradation of two well-known ADAMTS-5 substrates such as brevican [39] and aggrecan [40] facilitate tumor progression in glioblastoma and laryngeal carcinoma, respectively. Of note, ADAMTS-4 is expressed at lower level than ADAMTS-5 in tumors of the larynx [40] and in the metastatic loci of head and neck cancers [41]. This could indicate that ADAMTS-4 exerts a less relevant role than ADAMTS-5 in the metastatic processes of certain tumor types. Moreover, our results also indicate that Fibulin-2 is not a substrate of ADAMTS-1. Interestingly, it has been previously shown that Fibulin-1, the closest partner of Fibulin-2, is not a substrate but it can act as a cofactor of ADAMTS-1 [42]. As happens with most of the members of the family, ADAMTS-1 behaves as protumor or tumor-suppressor metalloprotease depending on different factors. For instance, ADAMTS-1 functions as a protumor protease if it is produced by stroma-derived cells, but not when produced by tumor cells [43]. In addition, anti-angiogenic activity associated to ADAMTS-1 promotes inhibition of liver metastasis but not lung metastasis [44]. Consequently, interactions between ADAMTSs and fibulins could be an additional feature that influence the different behavior displayed by ADAMTSs depending on the tissue or cell type where they are produced. Also, it remains to know whether Fibulin-2 could also act in a similar manner than Fibulin-1 does on ADAMTS-1 activity.

We have also explored the consequences of Fibulin-2 degradation within the tumor extracellular matrix. In fact, Fibulin-2 can act as a physical barrier hampering the dissemination of cancer cells in breast tumors and thereby loss of its expression has been associated with progression of cancer cells [12]. Our data using the poorly invasive breast cancer cell lines MCF-7 and T47D point out that protective role of Fibulin-2 could also be abolished through its degradation by ADAMTS-5. This effect would be especially relevant it those tissues containing high levels of Fibulin-2. In this regard, immunostaining of breast cancer tissues samples underlines the close proximity or partial co-localization of Fibulin-2 and ADAMTS-5 in different areas of grade-III invasive ductal carcinomas. Moreover, ADAMTS-5 can also be detected at the periphery of Fibulin-2, thus allowing to speculate that Fibulin-2 proteolytic fragments could increase their solubility making their detection more difficult. Western blot analysis using a set of normal-tumor paired samples also revealed that ADAMTS-5 is highly expressed in the tumor samples analyzed. Similarly, Gu *et al*. [45] have recently found that ADAMTS-5 is highly expressed in non-small cell lung cancer samples as compared with their paired adjacent non-tumor tissue. This elevated expression was associated with an enhancement of migration and invasion of malignant cells in this type of tumors, and with a poor prognosis of the affected patients. These data would indicate that ADAMTS-5 may act as metastatic promoter in tumors of different origins. In reference to Fibulin-2, our results indicate that is mainly detected in normal breast tissue, as has been previously reported by Yi *et al*. [12]. It is noticeable that some samples showed that Fibulin-2 is proteolytically processed, which can be due to the activity of ADAMTS-5 attending to the presence of a immunoreactive band of approximately 50 kDa. However, it cannot be ruled out that other enzymes also contribute to degrade Fibulin-2 in tumor processes [15]. For instance, gelatinases MMP-2 and MMP-9 are highly associated with tumor progression and both can effectively cleave Fibulin-2 [18]. In this work we have compared Fibulin-2 digestion pattern generated by MMP-2 with that produced by ADAMTS-5 activity concluding that Fibulin-2 might be a substrate for both metalloproteases in breast cancer. Interestingly, it has been widely shown that MMPs and ADAMTSs share the ability to cleave other extracellular components as is the case of aggrecan. In fact, cartilage aggrecan can be degraded within the interglobular domain but with different specificities and efficiencies [46]. It is also worth to mention that other factors can influence the activity of ADAMTS-5 on Fibulin-2 as can been the presence of TIMPs. In special, TIMP-3, a potent inhibitor of ADAMTS-5 [47], has been previously identified in breast cancer samples [48]. Consequently ADAMTS-5 could remain inactivate despite of its high expression in some tumor tissues samples. These extracellular matrix components and their biological effects highlight the molecular complexity and heterogeneity of the tumor microenvironment.

We also wanted to evaluate whether a potential degradation of Fibulin-2 by ADAMTS-5 could promote other changes in the tumor microenvironment. To this end we generated fibroblast spheroids, which have been successfully employed to investigate different aspects concerning the crosstalk between tumors cells and their surrounding stroma [49–51]. We found that that conditioned medium from MCF-7 cells overproducing both extracellular matrix proteins clearly induces an increase of the migratory and invasive properties of mammary fibroblasts when cultured as spheroids in 3D collagen matrices as compared with the effect of conditioned medium from MCF-7 overproducing Fibulin-2 alone. This pro-invasive effect was similar but less pronounced than that for spheroids cultured in the presence of conditioned medium from MCF-7 cell overproducing ADAMTS-5 alone. This result could also indicate that ADAMTS-5 exhibits a potent chemotactic activity to recruit fibroblasts as has been previously demonstrated for ADAMTS-1 [44]. In this sense, we observed an increase in the α-SMA levels in mammary fibroblasts when exposed to conditioned medium from MCF-7 overproducing Fibulin-2 and ADAMTS-5, effect that can be related to a fibroblast activation. Thus, it can be inferred that simultaneous presence of Fibulin-2 and ADAMTS-5 can actively modify cellular behavior of breast cancer cells as well as the surrounding mammary fibroblasts, which represent a major and crucial component of tumor stroma. In this work we have also found that ADAMTS-12 can inhibit Fibulin-2 degradation. ADAMTS-12 is mainly produced by stromal cells at the earliest stages not only of breast tumors [31] but also of colon cancer [52, 53]. Our new data support that the protection that ADAMTS-12 exerts on Fibulin-2 degradation by aggrecanases might form part of a defensive response against tumor invasion by strengthening the physical barrier created by Fibulin-2 and its interacting partners. Consequently, a decrease in expression levels of ADAMTS-12 in more advanced stages of the disease, such as those analyzed in this work, would contribute to the spreading of tumor cells.

More functional and clinical studies are needed to fully understand the participation of ADAMTS-5 in tumor processes. Thus, it would be crucial to distinguish processes in which ADAMTS-5 acts as an antitumor protein through its angio-inhibitory activity [54], from those protumor actions in which its proteolytic function seems to be crucial. Also, it remains to investigate whether any Fibulin-2 proteolytic fragment promotes bioactive functions as happens with versikine, a fragment containing the N-terminal G1 domain of versican and that is generated by the proteolytic activity or certain ADAMTSs including ADAMTS-5 [55]. In the case of Fibulin-2, a potential bioactive fragment should comprise a region near the N-terminus of the protein attending to the localization of the epitope (mapping between amino acids 28 and 277). These future challenges will help to determine how the relationship between Fibulin-2 and ADAMTS metalloproteases could also influence the tumor-protective role associated to Fibulin-2 in other types of tumors such as nasopharyngeal carcinomas [11]; or the tumor-promoting functions of Fibulin-2 in lung cancer [10]. In the meantime, degradation or protection of Fibulin-2 by ADAMTS metalloproteases provide new insights into the molecular mechanisms governing cellular microenvironment in normal and pathological conditions.

## MATERIALS AND METHODS

### Cell lines and cell culture conditions

Human breast cancer cell lines MCF-7, T47D and the human embryonic kidney cell line HEK 293 were kindly provided by Dr. Carlos Lopez-Otin, Universidad de Oviedo, Spain. SK-BR-3 was obtained from ATCC. Cells were cultured in DMEM supplemented with 10% fetal bovine serum, 50 μg/mL streptomycin and 100 U/mL penicillin (Life Technologies). Human mammary fibroblasts were purchased from Innoprot. For transfection experiments, vectors containing full-length human cDNA for ADAMTS-1 (Origene, ref. RC205984), ADAMTS-4 (Origene, ref. RC209226), ADAMTS-5 (Origene, ref. RC210834) and Fibulin-2 (cDNA clon HK9, kindly provided by Dr. T. Sasaki, Oita University, Japan) [3] were transfected into cells at 70–80% confluence using TransIT-X2 Dynamic Delivery System (Mirus). Cells stably producing recombinant proteins were selected in the presence of 500 μg/ml of geneticin (Sigma-Aldrich).

### Western blot analysis

For western blot analysis, cell extracts were resolved by 10% gel electrophoresis and transferred to polyvinylidene difluoride (PVDF) membranes (Millipore). When indicated, recombinant ADAMTS-1, ADAMTS-4 and ADAMTS-5 were detected using a primary anti-FLAG antibody (Sigma-Aldrich). Anti-Fibulin-2 (H-250) and anti-ADAMTS-5 (H-200) antibodies were from Santa Cruz Biotechnology, and anti ADAMTS-12 was from Proteintech. Anti-β-actin, Anti-β-tubulin and anti-α-SMA antibodies were from Sigma-Aldrich. In the case of HEK293 cells, 1 ml of conditioned medium was precipitated with four volumes of cold acetone, placed in freezer for 1 hour, centrifugated at 1000 × g for 10 min, and the pellet was resuspended in 2× SDS-PAGE loading buffer. Immunoreactive proteins were visualized using HRP-peroxidase labeled anti-rabbit or anti-mouse secondary antibodies and the ECL detection system (Pierce).

### Invasion assay

Invasion potential of T47D and MCF-7 cells was evaluated using 24- well Matrigel-coated invasion chambers with a 8 μm pore size (BD Biosciences) and at least three independent experiments were performed for each condition. To this end, 1.2 × 10^5^ cells were allowed to migrate for 96 h using 10% foetal bovine serum as a chemoattractant. Cells that reached the lower surface were fixed with 4% paraformaldehyde and stained with crystal violet. Total number of cells in the lower chamber was determined by visible light microscopy (LAS EZ 2.0 Leica, Microsystems). In the case of SK-BR-3 cells, invasion assay was performed as above but effect of the exogenous expression of ADAMTS-12 in cell migration was also probed.

### Enzymatic assay

Proteolytic assay on Fibulin-2 (0.5 μg per reaction, kindly provided by Dr. T. Sasaki, Oita University, Japan; or purchased from Abnova, ref. 28–1184), was carried out in 50 μL of reaction buffer (50 mM Tris-HCl, 150 mM NaCl, 10 mM CaCl_2_, 0.05% Brij 35, pH 8.5). Recombinant ADAMTS-1, ADAMTS-4, ADAMTS-5 and MMP-2 were purchased from R&D Systems and employed at 50 nM. Reactions were allowed to proceed at 37°C for the indicated times and stopped by addition of reducing SDS-sample buffer containing 20 mM EDTA. Then, digestion products were visualized by Western blot as described above. When indicated, samples were preincubated with ADAMTS-12 for 30 min before addition of ADAMTS-5. Production and purification of recombinant ADAMTS-12 was performed as previously described [56].

### Three dimensional sphere invasion assay

Human mammary fibroblasts were suspended in DMEM plus 5% Methyl cellulose (Sigma) at 2000 cells/ 25 μl medium. Cell spheroids were subsequently obtained by serial pipetting of 25 μl into a non-adhesive bacterial Petri dish (2000 cells/ spheroid) and incubated in an inverted position for 18 hours. Next day, each cell spheroid was transferred to an individual well of a 96-well plate and embedded into 110 μL of 2.3 mg/mL bovine collagen matrix (Advanced Biomatrix PureCol). After two hours polymerization at 37°C, each well was filled with 100 μL of control medium or the indicated conditioned medium. Collective cell invasion was monitored using a Zeiss Cell Observer Live Imaging microscope (Zeiss, Thornwood, NY) coupled with a CO_2_ and temperature-maintenance system. Time-lapse images were acquired every 30 min during 20 h using a Zeiss AxioCam MRc camera. The invasive area was determined by calculating the difference between the final area (*t* = 20 h) and the initial area (*t* = 0 h) using image J analysis program.

### Breast tissue samples

A breast tissue array containing 48 tumors samples of invasive ductal breast carcinoma from different tumor stages and three normal breast tissues was employed to examine the localization of ADAMTS-5 and Fibulin-2 in human breast samples. Histological diagnosis was carried out by an experienced pathologist and all protocols were approved by the Ethics Committee of the Hospital Universitario Central de Asturias for the project PI11/2011. Written informed consent was obtained from the patients. Slides were processed for indirect peroxidase immunohistochemistry. To this end, sections were deparaffinized and rehydrated, and then rinsed in phosphate buffered saline (PBS) containing 1% tween-20. Santa Cruz Biotechnology antibodies were used for the detection of Fibulin-2 (H-250) and ADAMTS-5 (D-16), and samples were heated in high pH Envision FLEX target retrieval solution at 80°C for 20 min and then incubated for 20 min at room temperature in the same solution. Endogenous peroxidase activity (3% H_2_O_2_) and non-specific binding (33% fetal calf serum) were blocked and the sections were incubated overnight at 4°C with primary antibodies described above using a 1:100 dilution for both antibodies. As secondary antibodies we used labeled polymer-HRP ready to use from DAKO. 3-3′ diaminobenzidine was employed as a chromogen. Selected slides were counterstained with haematoxylin. For fluorescence microscopy, serial sections, 8 μm thick, from IDC grade III, were processed for double immunofluorescence using primary antibodies against Fibulin-2 and Alexa 448-conjugated goat anti-rabbit IgG secondary antibodies (BioRad, 1:500 dilution). In the case of ADAMTS-5, incubation with primary antibody was followed by a bridge mouse anti-rabbit IgG antibody (Thermo Fisher Scientific, 1:100 dilution) and then by a CyTM3-conjugated donkey anti-mouse secondary antibody from Jackson-ImmunoResearch (1:100 dilution). In addition, nuclei were stained with DAPI (4′,6-diamidino-2-phenylindole) to ascertain structural details. Specificity of immunoreactivity was tested by omitting the primary antibodies or by replacing them with a non-immune serum.

Six breast tumor samples and a set of eight pairs of matched normal and tumor breast cancer tissue samples were also analyzed by western blot. To this end, samples were homogenized in a buffer containing 20 mM Tris-HCl, pH 7.4, 150 mM NaCl, 1% Triton X-100 (Sigma-Aldrich), 10 mM EDTA and Complete protease inhibitor cocktail (Applied Science). Tissue extracts were centrifuged at 15,000 × g at 4°C, supernatant collected and protein concentration was determined by the BCA protein assay kit (Pierce Biotechnology). Then, 20 μg of protein was resolved by gel electrophoresis and transferred to polyvinylidene difluoride (PVDF) membranes as indicate above. Staining of PVDF membranes with Ponceau S solution (Sigma-Aldrich) was employed to examine protein loading.

### Statistical analysis

Data were analyzed using Microsoft Excel and represented as means +/− S.E. Significant differences were determined with the Student *t* test and *p* values under 0.05 were considered statistically significant (*p* < 0.05, *p < 0.01, **p < 0.005, ***).

### Abbreviations

ECM; extracellular matrix; MMP, matrix metalloproteinase; ADAMTS, a disintegrin and metalloprotease with thrombospondin motifs; TIMP, tissue inhibitor of metalloprotease; α-sma, alpha smooth muscle actin.

## SUPPLEMENTARY MATERIALS


