# Supplementary material for: Cleavage of Fibulin-2 by the aggrecanases ADAMTS-4 and ADAMTS-5 contributes to the tumorigenic potential of breast cancer cells
Source: Oncotarget. 2017 Jan 11;8(8):13716–29. doi: 10.18632/oncotarget.14627 (PMC5355132; doi:10.18632/oncotarget.14627)
Supplement: Supplementary file 1 [file oncotarget-08-13716-s001.pdf]

Cleavage of Fibulin-2 by the aggrecanases ADAMTS-4 and ADAMTS-5 contributes to the tumorigenic potential of breast cancer cells

Supplementary Materials

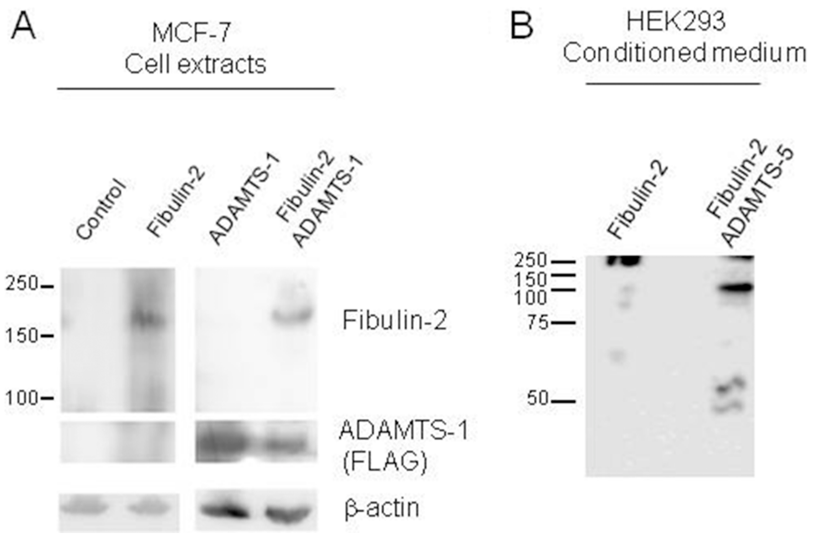

Supplementary Figure 1: (**A**) ADAMTS-1 does not cleave Fibulin-2 in MCF-7 cells. MCF-7 cells were transfected with a vector containing the full-length cDNA for Fibulin-2, ADAMTS-1 (tagged with a FLAG-epitope) or co-transfected with cDNAs for ADAMTS-1 and Fibulin-2. Control indicates cells transfected with an empty vector. Molecular weights are indicated on the left. β-actin was used as loading control. (**B**) Detection of Fibulin-2 proteolytic fragments in conditioned medium from HEK293 cells. HEK293 cells were transfected with a cDNA for Fibulin-2 or co-transfected with cDNAs for F ibulin-2 and ADAMTS-5, conditioned medium was concentrated and Fibulin-2 degradation products analyzed using an anti-Fibulin-2 antibody. Molecular weights markers are indicated on the left.

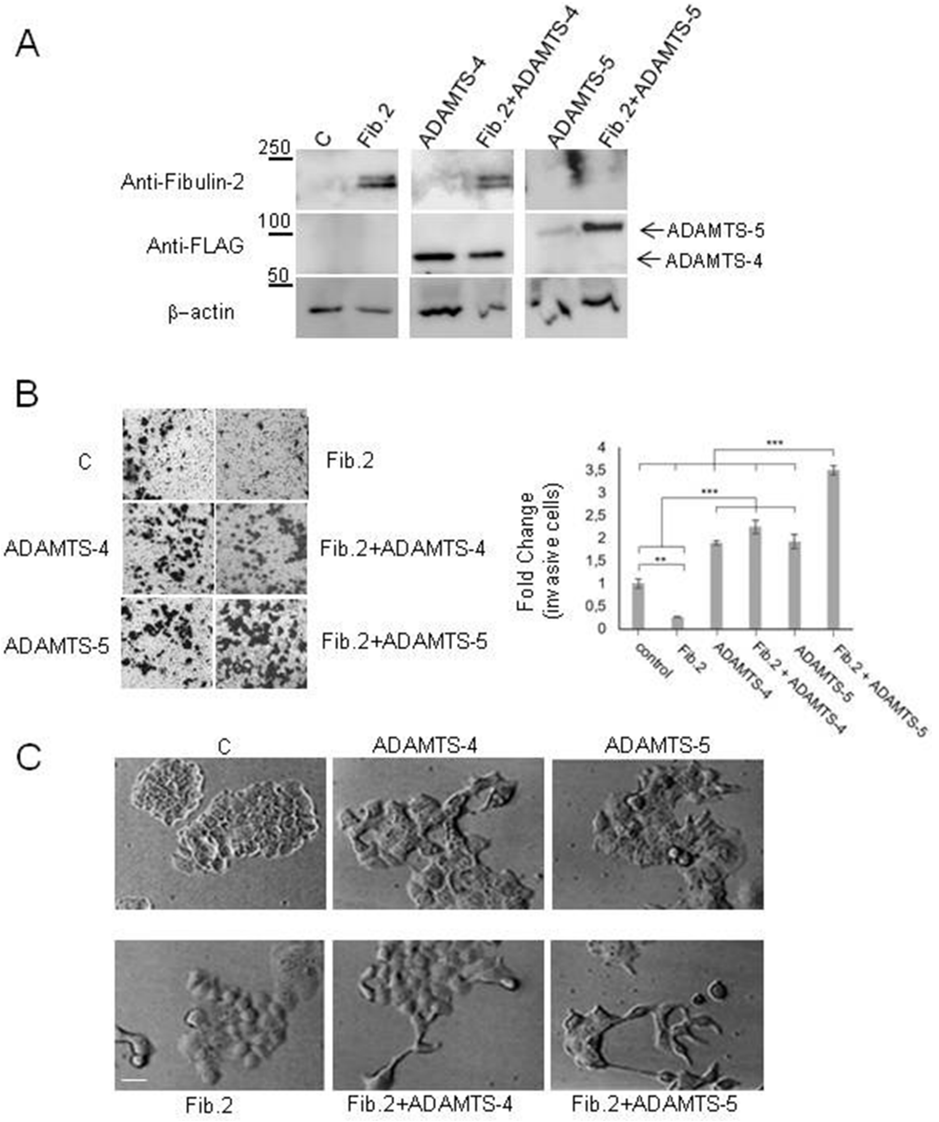

Supplementary Figure 2: Fibulin-2 influences invasion capacity of T47D depending on aggrecanases. (**A**) Western blot analysis of T47D cells expressing exogenous Fibulin-2 (Fib.2), ADAMTS-4, Fibulin-2 and ADAMTS-4 simultaneously (Fib.2 + ADAMTS-4), ADAMTS-5, or Fibulin-2 and ADAMTS-5 simultaneously (Fib.2 + ADAMTS-5). C indicates control cells transfected with an empty vector. Fibulin-2 was detected using a specific antibody and metalloproteases ADAMTS-4 and ADAMTS-5 were detected using an anti-FLAG antibody. Intervening irrelevant lanes are not shown. Molecular weight markers are indicated on the left and β-actins were used as loading control. (**B**) Left, representative pictures showing invasive T47D cells using Matrigel-coated invasion chambers producing the indicated exogenous proteins. C, control cells. Right, quantification of fold-change in invasion capacity of T47D cells under the different conditions assayed as compared with control cells (relative values). (**C**) Representative pictures of T47D cells expressing the indicated extracellular proteins. Simultaneous presence of Fibulin-2 and ADAMTS-5 induces visible morphological changes related to an epithelial-mesenchymal transition. Bar indicates 20 μm.

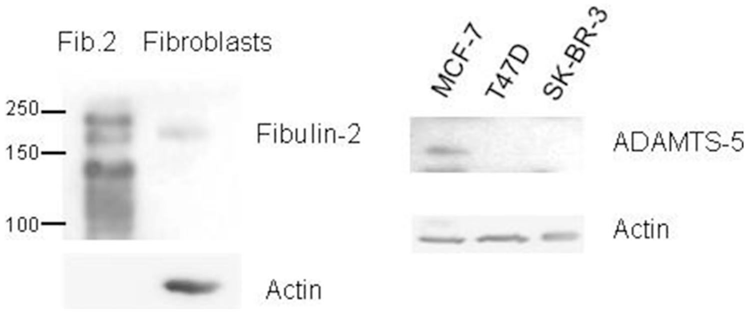

Supplementary Figure 3: Analysis of the endogenous expression of Fibulin-2 in mammary fibroblasts and of ADAMTS-5 in breast cancer cell lines. Left, analysis of the endogenous expression of Fibulin-2 by western blot was assessed in the normal breast fibroblasts employed in this work (Fib.2 indicates recombinant Fibulin-2 alone). Molecular weight markers are indicated on the left. Right, endogenous expression of ADAMTS-5 in the breast cancer cell lines employed in this study. β-actins were used as loading control.

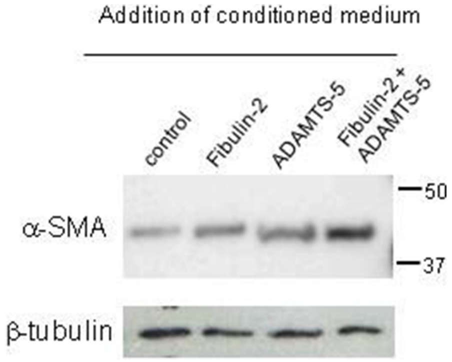

Supplementary Figure 4: Conditioned medium from MCF-7 cells overexpressing Fibulin-2 and ADAMTS-5 increases the α-SMA levels in normal breast fibroblasts. Normal breast fibroblasts were incubated with conditioned medium from MCF-7 cells transfected with cDNAs as indicated in the figure. Control refers to conditioned medium from MCF-7 cells transfected with an empty vector. Molecular weight markers are indicated on the right and β-tubulin levels were employed as loading control.

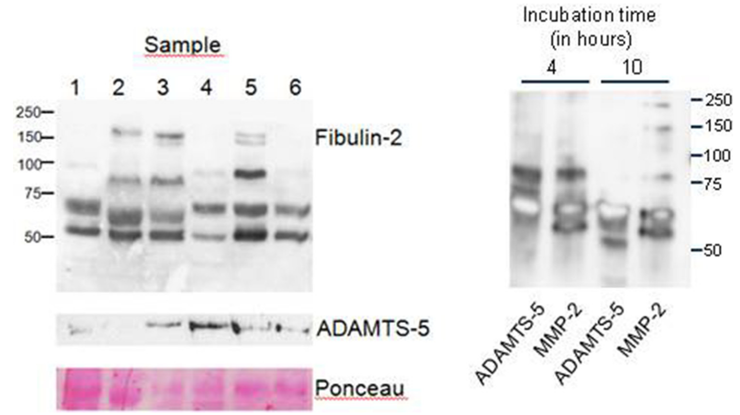

Supplementary Figure 5: Detection of Fibulin-2 proteolytic fragments in breast tumor samples or following the *in vitro* degradation by ADAMTS-5 or MMP-2. Left, detection of Fibulin-2 proteolytic products by western blot (molecular weight markers are indicated on the left) in six breast tumor samples. Presence of ADAMTS-5 is also analyzed. Ponceau S staining was employed to assess protein loading. Right, *in vitro* digestion of Fibulin-2 by ADAMTS-5 or MMP-2 at 4 and 10 hours as indicated. Cleavage products were detected using same antibody employed to analyzed breast cancer samples. Molecular weight markers are on the right.

Supplementary Video 1: Human mammary spheroid exposed to conditioned medium from control MCF-7 cells (control condition, control_V1). See Supplementary_Video_1

Supplementary Video 2: Human mammary spheroid exposed to conditioned medium from MCF-7 cells transfected with an empty vector (WT_V2). See Supplementary_Video_2

Supplementary Video 3: Human mammary spheroid exposed to conditioned medium from MCF-7 cells transfected with a vector containing the full-length cDNA for Fibulin-2 (Fib.2_V3). See Supplementary_Video_3

Supplementary Video 4: Human mammary spheroid exposed to conditioned medium from MCF-7 cells transfected with a vector containing the full-length cDNA for ADAMTS-5 (ADAMTS-5_V4). See Supplementary_Video_4

Supplementary Video 5: Human mammary spheroid exposed to conditioned medium from MCF-7 cells transfected with a vector containing the full-length cDNA for Fibulin-2 and ADAMTS-5 (Fib.2+ADAMTS-5_V5). See Supplementary_Video_5
